# A Class I HDAC Inhibitor BG45 Alleviates Cognitive Impairment through the CaMKII/ITPKA/Ca^2+^ Signaling Pathway

**DOI:** 10.3390/ph15121481

**Published:** 2022-11-28

**Authors:** Jingyun Liu, Chenghong Zhang, Jiale Wang, Yufei Huang, Di Shen, Yingqiu Hu, Haiying Chu, Xuebin Yu, Liyuan Zhang, Haiying Ma

**Affiliations:** Department of Histology and Embryology, College of Basic Medical Sciences, Dalian Medical University, Dalian 116044, China

**Keywords:** Alzheimer’s disease, proteomics, histone deacetylase inhibitor, synaptic plasticity

## Abstract

Alzheimer’s disease (AD) seriously endangers the health and life of elderly individuals worldwide. However, despite all scientific efforts, at the moment there are no effective clinical treatment options for AD. In this work, the effect of the class I histone deacetylase inhibitor (HDACI) BG45 on synapse-related proteins was investigated in primary neurons from APP/PS1 transgenic mice. The results showed that BG45 can upregulate the expression of synaptotagmin-1 (SYT-1) and neurofilament light chain (NF-L) in primary neurons. In vivo, the APPswe/PS1dE9 (APP/PS1) transgenic mice were treated with BG45 (30 mg/kg) daily for 12 days. Behavioral testing of BG45-treated APP/PS1 mice showed improvements in learning and memory. BG45 can alleviate damage to the dendritic spine and reduce the deposition of Aβ. Similar to the in vitro results, synapse-related proteins in the prefrontal cortex were increased after BG45 treatment. Proteomic analysis results highlighted the differences in the biological processes of energy metabolism and calmodulin regulation in APP/PS1 mice with or without BG45 treatment. Further verification demonstrated that the effect of BG45 on synapses and learning and memory may involve the CaMKII/ITPKA/Ca^2+^ pathway. These results suggest that class I HDACI BG45 might be a promising drug for the early clinical treatment of AD.

## 1. Introduction

Alzheimer’s disease (AD) is the most common type of dementia. Although breakthroughs have been made in AD research, AD is still incurable. Symptoms are not obvious in the early stages of AD and as the disease progresses, AD is characterized by extracellular senile plaques (SPs) formed by Aβ deposition and the accumulation of hyperphosphorylated tau protein as neurofibrillary tangles (NFTs) [1]. Synaptic loss, gliosis, neuroinflammation, and vascular dysfunction may also occur [2]. Based on the classic amyloid cascade hypothesis, soluble Aβ oligomers are considered to be the most toxic species that damage synapses by disrupting normal synaptic function and triggering downstream toxic pathways [3,4], leading to cognitive impairment.

Loss of synapses and synaptic damage are strongly associated with cognitive deficits found in AD patients [5]. Therefore, the loss of dendritic spines and synapses has become the focus of research on central nervous system diseases. Synaptic pathology might occur early in AD [6], and synaptic loss could precede neuronal degeneration [7]. Decreased expression of pre- and postsynaptic proteins is a core feature of AD pathophysiology. Synaptic biomarkers may link synaptic degeneration and cognitive decline in clinical practice, especially in the preclinical AD stage [8]. The expression of related synaptic proteins decreases, followed by synaptic changes such as synapse loss and synaptic function impairment, suggesting that synapse-related proteins are involved in the regulation of synaptic plasticity which may eventually lead to the decline of memory ability. Therefore, rescuing synaptic damage and reducing synaptic loss in the early stage of AD may be key to alleviating memory and cognitive decline.

Studies have shown that cognitive deficits are observed in APPswe/PS1dE9 (APP/PS1) transgenic mice at the age of three months in the radial arm water maze (RAWM) spatial working memory task, and are also seen at six months of age in the Morris water maze (MWM) [9]. Cognition is a complex trait, and although diverse brain regions are involved, most cognitive processes appear to engage cortical regions [10]. The prefrontal cortex controls the highest level of cognitive processes, and dysregulation of synaptic transmission in the prefrontal cortex is associated with memory loss in brain diseases such as autism, schizophrenia, depression, and AD [11]. The above mentioned findings strongly support that the cortex is one of the brain regions most frequently involved in the characteristic pathological alterations associated with AD.

Epigenetic modifications, such as DNA methylation and histone acetylation, have opened a new avenue for AD research [12]. Histone acetylation loosens chromatin to activate transcription, and histone deacetylation represses gene transcription [13]. Eukaryotic HDACs are mainly divided into four categories, among which Class I HDACs include HDACs 1, 2, 3, and 8 [14]. In the pathology of AD, a modification of the balance between histone-acetyltransferases (HATs) and histone-deacetylases (HDACs) leads to aberrant acetylation. HDAC inhibitors (HDACIs) have been proven to be promising drugs in AD animal models, such as APP/PS1 mice, T2579 mice, and CK-p25 mice [15]. Recently, the role of Class I HDACIs in recovering cognitive function [16,17] in a model of neurodegeneration have been proven. Class I HDACIs may improve the decrease in dendritic spine density and synapse loss by inhibiting the expression of HDAC [18]. In our previous research, class I HDACI BG45 upregulated the levels of synaptic-related genes in the hippocampus of an Aβ-injected AD model by specifically inhibiting class I HDACs [19]. However, the role of BG45 in the cortex of AD mice is unclear. Different neurodegenerative disorder (NDD) experimental models have also confirmed the neuroprotective effects caused by HDACs inhibitor through the regulation of neuronal apoptosis, inflammatory response, and metabolic dysfunction [15,20].

Therefore, this study focused on changes in the prefrontal cortex region of APP/PS1 mice. We investigated the effect of class I HDACI BG45 on the expression of synapse-associated proteins and cytoskeletal proteins in primary cortical neurons of APP/PS1 mice, and further explored the protective effect of BG45 on synaptic damage in the prefrontal cortex region and behavioral changes in APP/PS1 mice. The correlation between BG45 and reduced synaptic damage in the cerebral cortex of AD mice with treatment was explained by the results of whole proteomic analysis.

## 2. Results

### 2.1. Identification and Purity of the Primary Cortical Neurons from APP/PS1 Transgenic Mice

**Figure 1:**
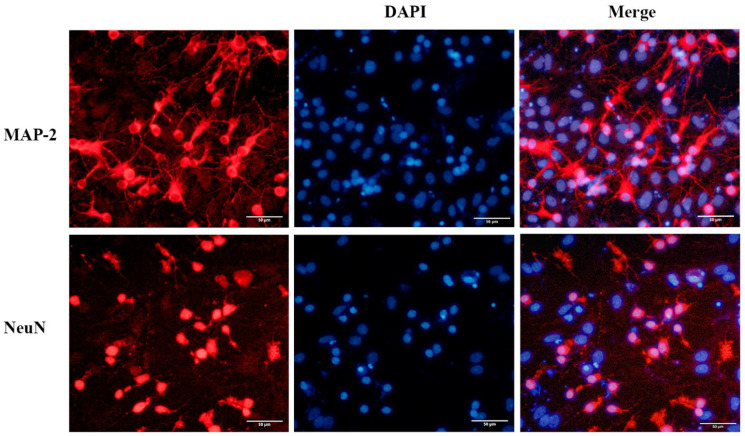
Immunofluorescence staining of primary cultured neurons from the neonatal mouse cortex. The neurons were immunostained to detect MAP-2 (red) and NeuN (red), and cell nuclei were stained blue with DAPI. Scale bar = 50 μm.

The newly seeded primary neurons were evenly suspended in medium. After 7 days of culture, the neuron soma was plump, and protuberances had extended and were interwoven into the network. Primary neurons were identified with the neuronal markers NeuN and MAP-2. Immunofluorescence staining revealed that approximately 70% of these cells were neurons, which could be used for subsequent experiments (Figure 1).

### 2.2. BG45 Increased the Expression Levels of SYT-1 and NF-L in Primary Cultured Cortical Neurons

SYT-1 is a presynaptic marker protein essential for fast, synchronous neurotransmitter release in hippocampal neurons [21]. NF-L is a cytoskeletal protein that is abundantly expressed in neuronal axons. 

**Figure 2:**
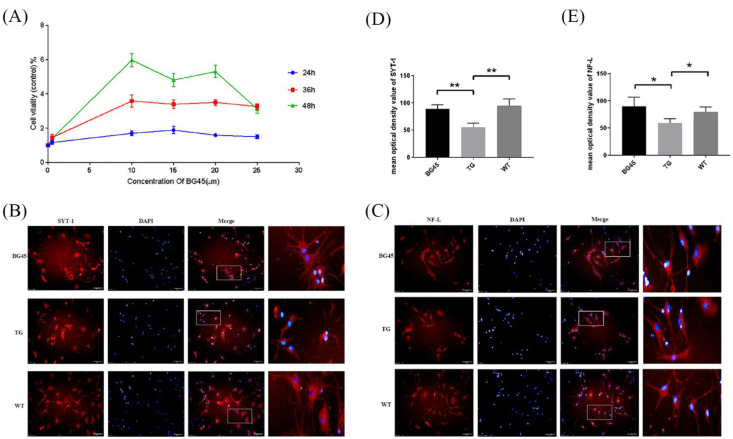
BG45 increased cell viability and the expression of SYT-1 and NF-L. (**A**) The viability of cells treated with BG45 (0.5, 10, 15, and 25 µM) for 24, 36, and 48 h. (**B**) Immunofluorescence staining analysis of SYT-1 (red) in primary neurons treated with BG45 (10 µM) for 48 h. (**C**) Immunofluorescence staining analysis of NF-L (red) in primary neurons treated with BG45 (10 µM) for 48 h. (**D**) Quantitative SYT-1 analysis. (**E**) Quantitative NF-L analysis. The values are presented as the mean ± SD from three independent experiments. * *p <* 0.05 and ** *p <* 0.01. Scale bar = 50 μm.

The optimal time and concentration of BG45 were screened by the CCK-8 method. The results showed that the viability of primary neurons reached the highest peak when 10 μM BG45 treatment was applied for 48 h. Therefore, 10 μM BG45 was applied for 48 h for subsequent experiments (Figure 2A).

Immunofluorescence staining showed that compared with the WT group, the SYT-1 and NF-L protein levels were significantly decreased in the TG group (*p* < 0.01, *p* < 0.05). Compared with the TG group, the SYT-1 and NF-L protein levels were significantly increased in the BG45 group (*p* < 0.01, *p* < 0.05; Figure 2B–E).

### 2.3. BG45 Improved Spatial Memory and Learning Ability in APP/PS1 Mice

**Figure 3:**
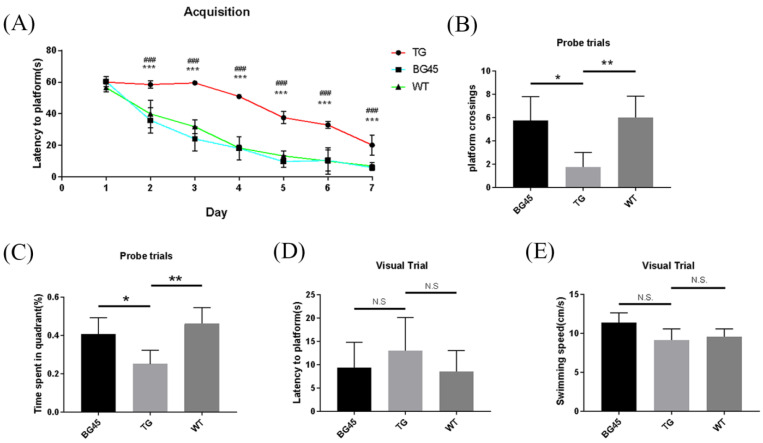
BG45 alleviated cognitive impairment in APP/PS1 transgenic mice. (**A**) Latency to platform (acquisition trial), (**B**) platform crossing (probe trial), (**C**) time spent in the target quadrant (probe trial), (**D**) latency to platform (visual trial), and (**E**) swimming speed. ^###^ *p <* 0.001: TG vs. BG45; * *p <* 0.05, ** *p <* 0.01, *** *p <* 0.001: TG vs. WT.

In this study, the Morris water maze test was used to evaluate spatial memory and learning ability of mice. In the acquisition trial, the learning curve showed that the time to reach the platform (escape latency) in the BG45 group was less than that in the TG group (*p* < 0.001; Figure 3A). In the probe test, the time spent in the target quadrant and the number of hidden platform crossings were significantly increased in the BG45 group (*p* < 0.05, *p* < 0.05) and the WT group (*p* < 0.01, *p* < 0.01), compared with the TG group (Figure 3B,C). To exclude the influence of visual differences and the swimming ability of mice in each group, visual platform trials and swimming speed tests were carried out. The results showed that there was no significant difference between these groups (Figure 3D,E).

### 2.4. Effects of BG45 on Dendritic Spines, the Expression of Synapse-Related Proteins and the Accumulation of Aβ in the Cortex of APP/PS1 Mice

**Figure 4:**
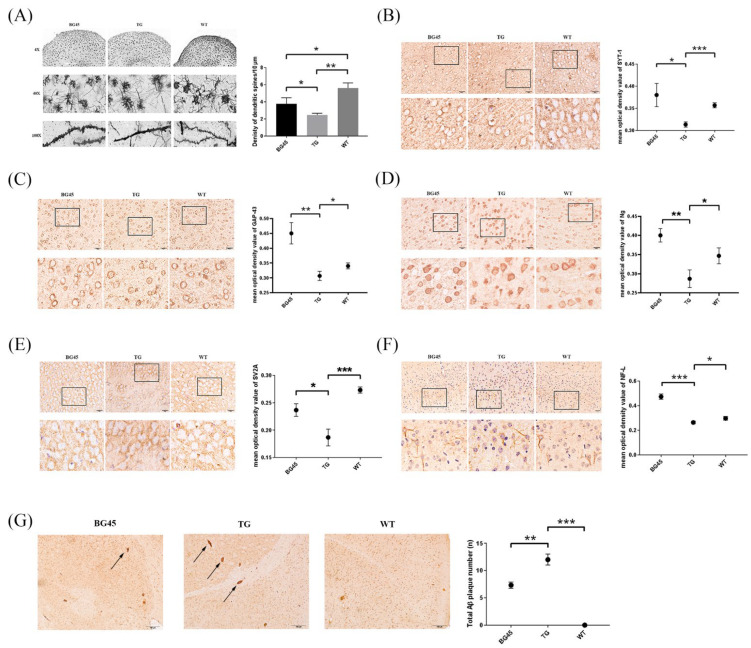
Effects of BG45 on the number of dendritic spines, the Aβ burden and the expression of synapse-related proteins in APP/PS1 mice. (**A**) Spine densities of Golgi-Cox-impregnated prefrontal cortex neurons in each group (scale bar = 10 μm) and quantification of spine density. (**B**) Immunohistochemistry analysis of SYT-1 in each group and quantification of SYT-1 expression. (**C**) Immunohistochemistry analysis of GAP-43 in each group and quantification of GAP-43 expression. (**D**) Immunohistochemistry analysis of Ng in each group and quantification of Ng expression. (**E**) Immunohistochemistry analysis of SV2A in each group and quantification of SV2A expression. (**F**) Immunohistochemistry analysis of NF-L in each group and quantification of NF-L expression (scale bar = 20 μm). The values are presented as the mean ± SD from three independent experiments. (**G**) The number of Aβ plaques in the prefrontal cortex area of the mice in each group (scale bar = 100 μm) and the quantification of Aβ deposition. * *p <* 0.05, ** *p <* 0.01, *** *p <* 0.001.

Golgi-Cox staining was used to evaluate the degree of dendritic spine loss in the prefrontal cortex of mice. The results showed that the numbers of dendritic spines in the prefrontal cortex of mice in the BG45 group (*p* < 0.05) and the TG group (*p* < 0.01) were less than that in the WT group. However, in the BG45 group, the number of dendritic spines was significantly recovered by BG45 compared with the TG group (Figure 4A).

Furthermore, the expressions of synapse-related proteins, which play a key role in dendritic spine’s shape and synapse plasticity, were detected by immunohistochemistry. Compared with the TG group, the expression levels of the synapse-related proteins SYT-1, growth-associated protein (GAP-43), neurogranin (Ng), and synaptic vesicle glycoprotein 2A (SV2A), and the cytoskeletal protein NF-L in the prefrontal cortex of the BG45 group were significantly increased (*p* < 0.05, *p* < 0.01, *p* < 0.01, *p* < 0.05, *p* < 0.001; Figure 4B–F).

When exploring the effect of BG45 on the pathological changes of AD in the prefrontal cortex of transgenic mice, the Aβ deposition in the prefrontal cortex in the BG45 group was significantly reduced compared with that in the TG group (*p* < 0.01; Figure 4G).

### 2.5. BG45 Decreased the Protein Expression Levels of HDAC1, HDAC2 and HDAC3 in the Cortex of APP/PS1 Mice

**Figure 5:**
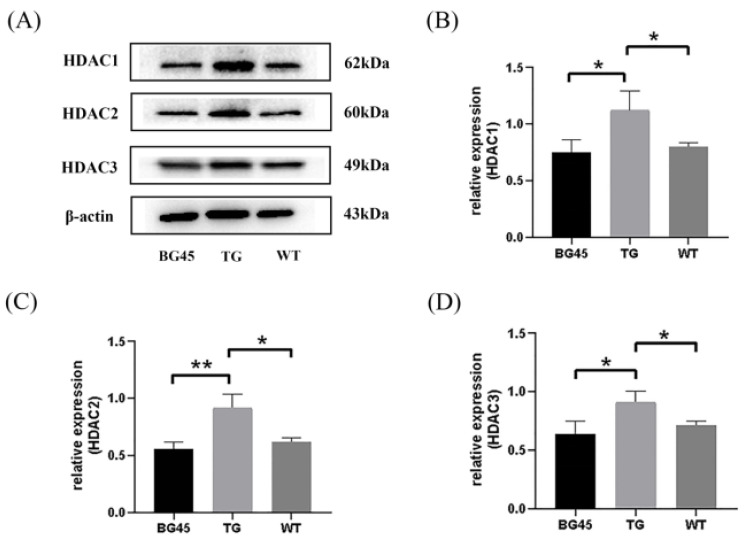
Effects of BG45 on class I HDACs (HDAC1, 2, and 3). (**A**) Representative protein expression bands of HDAC1, HDAC2 and HDAC3. (**B**) Quantification of HDAC1 expression. (**C**) Quantification of HDAC2 expression. (**D**) Quantification of HDAC3 expression. The values are presented as the mean ± SD from three independent experiments. * *p <* 0.05, ** *p <* 0.01.

As a class I HDAC inhibitor, the inhibition of BG45 on three subclasses was verified. The results showed that compared with the WT group, the protein levels of HDAC1, HDAC2, and HDAC3 in the TG group were increased (*p* < 0.05, *p* < 0.05, *p* < 0.05). Compared with the TG group, the protein levels of HDAC1, HDAC2, and HDAC3 in the BG45 group were decreased (*p* < 0.05, *p* < 0.01, *p* < 0.05; Figure 5).

### 2.6. Profiling of Differentially Expressed Proteins in the Prefrontal Cortex

**Figure 6:**
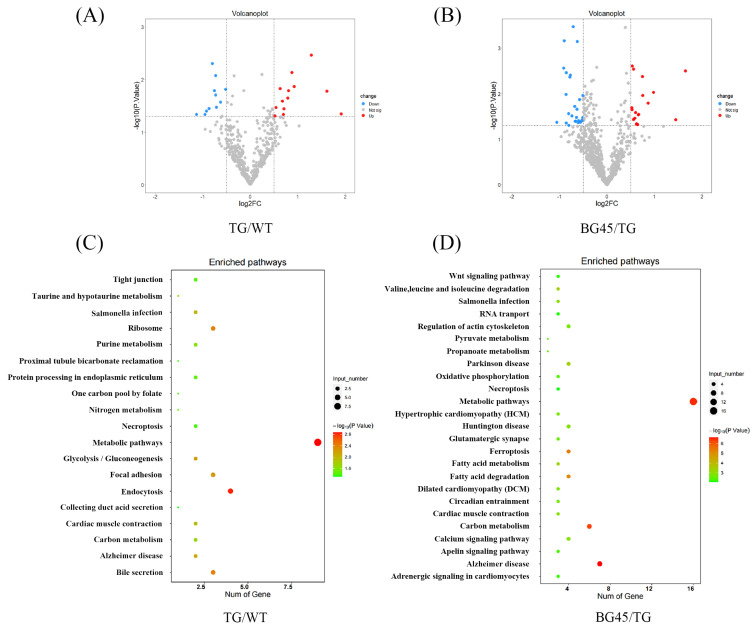
Differentially expressed proteins in the mouse prefrontal cortex and KEGG pathway analysis of differentially expressed proteins. (**A**) Volcano plot of differentially expressed proteins in the TG group vs. the WT group (red and blue points indicate up- or downregulated significant proteins, respectively). (**B**) Volcano plot of differentially expressed proteins in the BG45 group vs. the TG group (red and blue points indicate up- or downregulated significant proteins, respectively). (**C**) Pathway analysis of differentially expressed proteins in the TG group vs. the WT group. (**D**) Pathway analysis of differentially expressed proteins in the BG45 group vs. the TG group. P values are emphasized in color from the most extreme decrease (light green) to the highest increase (dark red).

To investigate the differences in the prefrontal cortex proteomes between each group, a proteomic analysis was conducted. From these data, 24 differentially expressed proteins between the TG and WT groups were identified, including 13 upregulated proteins and 11 downregulated proteins, and 45 differentially expressed proteins between the BG45 and TG groups, including 18 upregulated proteins and 27 downregulated proteins (Figure 6A,B). Pairwise comparison of the differentially expressed proteins between TG versus WT and BG45 versus TG showed that there were several overlapping differential proteins: CAII, Adh5, Capzb, Arhgef2, Cadm4, Gspt1, Gspt2, and ITPKA.

Pathway analysis based on the KEGG reference pathway databases (http://www.genome.jp/kegg/pathway.html, accessed on 10 December 2021) revealed that most of the significantly altered proteins between TG versus WT and BG45 versus TG were involved in Alzheimer’s disease and metabolism pathways (Figure 6C,D).

### 2.7. BG45 Influenced the CaMKII/ITPKA/Ca^2+^ Pathway In Vivo

**Figure 7:**
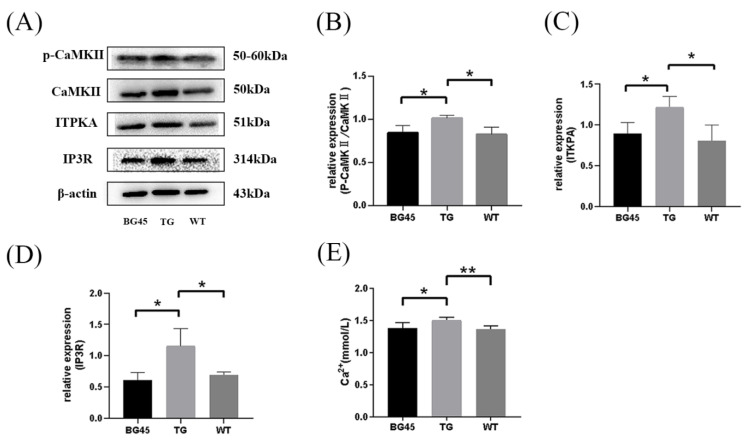
Effects of BG45 on p-CaMKII, CaMKII, ITPKA, IP3R and Ca^2+^. (**A**) Representative protein expression bands of p-CaMKII, CaMKII, ITPKA and IP3R. (**B**) Quantification of p-CaMKII/CaMKII analysis. (**C**) Quantification of ITPKA analysis. (**D**) Quantification of IP3R expression. (**E**) Impact of BG45 on serum calcium in APP/PS1 mice. The values are presented as the mean ± SD from three independent experiments. * *p <* 0.05, ** *p <* 0.01.

Based on the above proteomics analysis, the differential protein inositol-1,4,5-trisphosphate 3-kinase-A (ITPKA) was selected as the key point and validated several related proteins. Compared with the WT group, the protein expression levels of p-CaMKII, ITPKA and IP3R in the TG group were significantly increased (*p* < 0.05, *p* < 0.05, *p* < 0.05). Compared with the TG group, the protein expression levels of p-CaMKII, ITPKA and IP3R in the BG45 group were significantly decreased (*p* < 0.05, *p* < 0.05, *p* < 0.05; Figure 7A–D).

Consistently, it was found that the concentration of serum calcium in the TG group was significantly increased compared to that in the WT group (*p* < 0.01). Compared with the TG group, the concentration of serum calcium in the BG45 group was substantially decreased (*p* < 0.05; Figure 7E), which indicates that BG45 improves the imbalance of calcium dynamics in APP/PS1 mice.

## 3. Discussion

Alzheimer’s disease is one of the most common chronic neurodegenerative diseases. The role of class I HDACIs in treating AD pathology has been proven [16,19]. In this study, the effect of the class I HDACI BG45 on the AD model and the relevant mechanism of its neuroprotective effect were studied. The data proved that BG45 can improve the expression of synaptic plasticity-related proteins and cytoskeletal proteins in vitro and in vivo. It also demonstrated that BG45 can alleviate damage to dendritic spines, reduce the deposition of Aβ, and improve learning and memory in an AD model. The effects of BG45 on synapses, learning, and memory may be involved in the CaMKII/ITPKA/Ca^2+^ pathway. 

In this research, primary neurons in the cortex of APP/PS1 mice were harvested within seven days of birth. Studies have shown that primary neurons from APP/PS1 mice over secreted Aβ, and a significant reduction in cell viability was observed [22]. Another study revealed that class I HDACIs showed neuroprotective effects against Aβ toxicity in primary neurons [23]. In our research, cortical neurons were incubated with 10 µM BG45 for 48 h, and immunofluorescence was used to assay the expression of the synaptic vesicle protein SYT-1 and the cytoskeletal protein NF-L. Synaptotagmin-1 (SYT-1) has important functions in vesicle trafficking, docking, fusion to the synaptic plasma membrane, and neurotransmitter release [21,24]. SYT-1 triggers the release of neurotransmitters by combining with Ca^2+^ ions [25], affecting synaptic plasticity, learning, and memory. Neurofilaments (NFs) are structural scaffolding proteins and the major cytoskeletal component of most neurons. NFs consist of three major classes: the neurofilament light chain (NF-L), the neurofilament medium chain (NF-M), and the neurofilament heavy chain (NF-H). NF-L is the most abundant NF in axons, forming the core of the NF bundle [26]. It was found that the expression levels of SYT-1 and NF-L were decreased in primary neurons of APP/PS1 mice compared to WT mice, and BG45 upregulated the expression levels of SYT-1 and NF-L. The results demonstrated that the protective effect of BG45 on cortical neurons in APP/PS1 mice may be related to the upregulation of synaptic vesicle protein SYT-1 and cytoskeletal protein NF-L.

To further confirm the neuroprotection of BG45 in vivo, the effect of BG45 on prefrontal cortex synaptic damage and learning and memory ability in APP/PS1 mice were studied. The spatial exploration and learning abilities of the 6-month-old APP/PS1 mice were significantly decreased compared with those of the WT mice. However, in APP/PS1 mice treated with BG45 for 12 days, learning and memory impairments were obviously improved. Dendritic spines, which receive most of the excitatory synaptic input in the cerebral cortex, are heterogeneous with regard to their structure, stability, and function [27], and are thought to be the basis of brain plasticity. Synaptic loss is closely related to the loss of dendritic spines. Synapse and dendritic spine loss and dendritic atrophy are observed in many neurodegenerative diseases and psychiatric diseases, such as Alzheimer’s disease and major depressive disorder (MDD) [28]. These reductions in synaptic connectivity are believed to be a major contributor to impaired cognition [29]. It has been reported that the application of low-dose soluble Aβ oligomer can induce a decrease in the number of dendritic spines accompanied by morphological abnormalities and density reduction of dendritic spines [18,30]. Researchers found more severe loss of dendritic spines near plaques formed by beta-amyloid deposits [31]. This result indicated that Aβ may be one of the reasons for the reduction in dendritic spines. In our research, the number of dendritic spines in the prefrontal cortex of 6-month-old APP/PS1 mice was significantly lower than that in WT mice. BG45 treatment significantly improved the number of dendritic spines in APP/PS1 mice. A large number of Aβ plaques appeared in the prefrontal cortex of the TG mice; however, Aβ plaques in the prefrontal cortex of the BG45-treated mice were significantly reduced. Therefore, it is hypothesized that BG45 can alleviate synapse loss in the prefrontal cortex and improve learning and memory by increasing the number of dendritic spines and reducing Aβ plaques in APP/PS1 mice.

To further confirm these findings, several key proteins involved in synaptogenesis were examined. Growth-associated protein (GAP-43) is an important presynaptic membrane protein that is an activator of various enzymes involved in the activation of the axonal growth process, endocytosis/exocytosis recycling, and neurotransmitter transmission and is considered an intrinsic determinant of growth competence in neurons [32]. Synaptic vesicle glycoprotein 2A (SV2A) is widely expressed in neurons of the central nervous system and serves as a promising biomarker of synaptic density [33]. Recent studies using SV2A PET imaging have demonstrated significant synaptic loss in the hippocampus of AD patients [34]. Neurogranin (Ng) is a postsynaptic protein that is concentrated in the cell bodies and dendrites of neurons in the cerebral cortex and hippocampus [35]. Recent studies have demonstrated that Ng is involved in synaptic plasticity and synaptic regeneration mediated by the calcium- and calmodulin-signaling pathways [35,36]. In an inducible mouse model of neurodegeneration, the expression of Ng was significantly lower than that in normal mice [37]. Plasma Ng levels in AD patients are lower than those in normal controls, and are associated with the progression of mild cognitive impairment (MCI) to AD [38]. In this study, it was found that the protein expression levels of SYT-1, GAP-43, Ng, SV2A, and NF-L in the prefrontal cortex of APP/PS1 mice were significantly decreased compared with those in the WT group. What is exciting is that BG45 promotes the expression of these synaptic-related proteins. These results suggest that BG45 may protect against synaptic damage in APP/PS1 transgenic mice by upregulating synaptic-related proteins.

Since there is evidence that HDAC2 inhibitors significantly downregulate HDAC2 and upregulate the expression of synaptic plasticity genes in SH-SY5Y cells over-expressing amyloid precursor protein (SH-APP cells) [39], the inhibitory effect of the class I HDACI BG45 on various subclasses was examined in prefrontal cortex of the APP/PS1 mice in the study. The data showed that BG45 effectively reduced the expression levels of HDAC1, HDAC2, and HDAC3, consistent with the upregulation of the expression of synapse-associated proteins, including SYT-1, GAP-43, Ng, SV2A, and NF-L. Therefore, it is proved that BG45 improves synaptic proteins possibly by downregulating the expression of HDAC1, HDAC2, and HDAC3.

To further explore the underlying molecular mechanisms of BG45 in rescuing synaptic damage, a detailed proteomic functional analysis was performed, including KEGG pathway analysis, for the related potential proteins in the three groups. Our results showed that some of the main pathways identified were involved in Alzheimer’s disease, metabolism pathways and carbon metabolism. Among the eight proteins significantly influenced by BG45, ITPKA, which plays an important role in metabolic pathways, was identified for further exploration.

Inositol-1,4,5-trisphosphate 3-kinase-A (ITPKA) is a subtype of inositol 1,3,4-trisphosphate 5/6 kinase (ITPK) that is mainly expressed in the brain and is closely related to the developmental stage of the brain [40]. ITPK is highly expressed in hippocampal CA1 pyramidal and dentate gyrus granule cells [41]. The above mentioned results and other evidence [40] strongly support that ITPK may be involved in brain development and memory processes. Spatial learning enhances the expression of ITPKA in rats, suggesting that ITPKA plays a role in the processing of spatial memory, most likely by regulating calcium signaling in dendritic spines [42]. ITPK in mammals is regulated by Ca^2+^/CaM, protein kinase A (PKA), protein kinase C (PKC), calcium/calmodulin-dependent protein kinase II (CaMKII), and other regulatory factors. ITPK is positively regulated by CaMKII, and phosphorylation of CaMKII at Thr-311 increases ITPK activity 8-10-fold [43]. In the phosphoinositide cycle, ITPKA phosphorylates inositol 1,4,5-trisphosphate (IP3) at the 3′ position, thereby producing inositol 1,3,4,5-tetraphosphate (IP4). Both IP3 and IP4 are important second messengers. The combination of IP3 and IP3 receptor (IP3R) opens the channel releasing Ca^2+^ from the endoplasmic reticulum into the cytoplasm, which leads to an increase in intracellular free Ca^2+^, activating the calcium signaling pathway. The production of IP4 increases the half-life of IP3 [44] and cooperates with IP3 to mediate Ca^2+^ through the plasma membrane and regulate intracellular Ca^2+^ signaling [45,46]. In our case, the levels of p-CaMKII/CaMKII, ITPKA, and IP3R in the prefrontal cortex of APP/PS1 mice were significantly higher than those in WT mice, and the serum Ca^2+^ level was also significantly higher than that in the WT group. These results confirmed that the abnormal metabolism of phosphoinositide in transgenic mice leads to an imbalance in Ca^2+^ homeostasis. In the BG45 group, the levels of p-CaMKII/CaMKII, ITPKA, and IP3R were significantly decreased compared with those in the TG group, and the serum Ca^2+^ level was also significantly decreased. Studies have shown that the expression levels of GAP-43 [47], SYT-1 [48], SV2A [49], Ng [50], and NF-L [51] can be regulated by Ca^2+^; thus, it is speculated that BG45 may upregulate synapse-related proteins through the CaMKII/ITPKA/Ca^2+^ pathway.

## 4. Materials and Methods

### 4.1. Cell Cultures and Treatments

APP/PS1 (APPswe/PSldE9) mice and wild-type (C57BL/6J) mice were purchased from Nanjing Institute of Biomedicine of Nanjing University. All animals were housed at a temperature of 21.0 ± 2 °C and allowed access to food and water ad libitum. All procedures were approved by the Institutional Animal Care and Use Committee of Dalian Medical University in Dalian, China.

Primary cortical neurons were harvested from APP/PS1 transgenic mice and C57BL/6J mice within 7 days of birth following a standard protocol [52]. Mice were decapitated, and cortices were dissected under an anatomical microscope by removing the meninges and blood vessels in DMEM (Gibco, Billings, MT, USA) containing 10% FBS (Gibco, USA). After digestion with trypsin (Gibco, USA) and DNase I (Solarbio, Beijing, China) for 15 min at 37 °C, the cell suspension was filtered through a 70 µm cell strainer and resuspended in neurobasal medium (Gibco, USA) with 2% B27 (Gibco, USA), 1% GlutaMAX Supplement (Keygene, Nanjing, China) and 1% penicillin/streptomycin (Biosharp, Hefei, China). After the cells were counted under a microscope by trypan blue, they were placed on poly-L-lysine-coated plates (Solarbio, China). The purity of the primary neurons was assessed by immunofluorescence staining with rabbit anti-NeuN antibody (1:200, Abcam, Cambridge, UK, ab177487) and rabbit anti-MAP2 antibody (1:250, Proteintech, Rosemont, IL, USA, 17490-1-AP) after 7 days of culture.

### 4.2. CCK-8 Assay

Cell viability was analyzed by a Cell Counting Kit-8 (Solarbio, China) following the manufacturer’s protocol. In brief, after primary neurons were treated with 0.5 μM, 10 μM, 15 μM, 20 μM, or 25 μM BG45 (Selleck, Houston, TX, USA, 926259-99-6) for 12 h, 24 h, and 48 h, then 10 μL CCK-8 reagent was added to each well and incubated at 37 °C for 1 h. The absorbance was measured at 450 nm using a microplate reader (Thermo Fisher Scientific, Waltham, MA, USA). Cell viability was expressed as the ratio of the absorbance of the treated group to that of the control group.

### 4.3. Immunohistochemistry

Cells were cultured on 12-well poly-L-lysine-coated slides (Solarbio, China) and treated as described above. The cell climbing slides were washed with PBS and fixed in an appropriate amount of 4% paraformaldehyde (PFA) at room temperature for 20 min. Then, they were washed with PBS and permeabilized with 0.1% Triton-X100 at room temperature for 30 min. After washing the slides with PBS, they were blocked with 5% BSA at room temperature for 30 min. All slides were incubated with one of the following primary antibodies overnight at 4 °C: rabbit NeuN (1:200, Abcam, ab177487), rabbit MAP2 (1:250, Proteintech, 17490-1-AP), rabbit SYT-1 (1:50, Proteintech, 14511-1-AP), and rabbit NF-L (1:50, Abcam, ab223343). After washing three times with PBS, the slides were incubated with fluorophore-labeled secondary antibody (1:300, Proteintech, CL594-10594) for 1 h at room temperature in a dark and moist environmental box. Then, DAPI was added to the samples at room temperature for 20 min. Cell images were captured by fluorescence microscopy (Olympus, Tokyo, Japan).

For the tissue, the prefrontal cortex region was cut into sections from paraffin blocks. After deparaffinization and antigen retrieval following standard procedures, the slides were added to the appropriate amount of endogenous peroxidase at room temperature for 15 min and blocked by incubation with goat serum albumin at room temperature for 20 min. The slides were then incubated with one of the following primary antibodies overnight at 4 °C in a moist environment box: rabbit Aβ_1-42_ (1:200, Cell Signaling Technology, Danvers, MA, USA, 24090), rabbit SYT-1 (1:100, Proteintech, 14511-1-AP), rabbit GAP-43 (1:200, Proteintech, 16971-1-AP), rabbit Ng (1:5000, Abcam, ab217672), rabbit SV2A (1:1000, Abcam, ab254351), and rabbit NF-L (1:4000, Abcam, ab223343). The slides were incubated with biotin-labeled goat anti-rabbit/mouse IgG at room temperature for 20 min. Afterward, an appropriate amount of streptavidin-HRP complex was added and incubated at room temperature for 15 min. After staining with DAB solution (ZSGB-Bio, Beijing, China), the slides were counterstained with hematoxylin for a few seconds and rinsed with 1% hydrochloric acid alcohol (Solarbio, China). Finally, the slides (*n* = 3/group) were observed using a optical microscope. The mean optical density was quantified by ImageJ software.

### 4.4. Animals and Drug Administration

Six-month-old male animals were divided into three groups: six-month-old APP/PS1 transgenic mice were randomly divided into the treatment group (BG45) and the non-treatment group (TG), and wild-type mice were used as the control group (WT). Five mice were included in each group. The BG45 group was intraperitoneally injected with BG45 (30 mg/kg) daily for 12 days [53], and the TG and WT groups were injected with the same amount of normal saline. After 12 days, the cognitive abilities of the mice were assessed by behavioral experiments, and all mice were killed within 24 h and samples were collected for different tests.

### 4.5. Morris Water Maze

The MWM mainly includes two parts: an acquisition trial and a probe trial. It also includes a visual trial and swimming speed tests. A double blind test was used in the experiment. In the acquisition test, each mouse was put into the water from different quadrants for 3 trials per day for 7 days. Mice were allowed to perform the test over a 60 s time span. If the mouse could not find the platform after 60 s, the experimenter used a guide stick to guide the mouse to find the platform, and placed the mouse on the platform for 20 s. The probe trial was performed 24 h after the last acquisition trial. Before the test began, the escape platform was removed from the water. Each mouse was put into the water from the quadrant diagonal to where the escape platform was located and allowed to swim freely for 60 s. A visual test was carried out immediately after the probe trial. The test system tracked the time each mouse found the platform and the swimming speed.

### 4.6. Golgi-Cox Staining

The morphology of dendritic spines was observed by Golgi-Cox staining (FD NeuroTechnologies, Inc., Columbia, MD, USA). All procedures followed the manufacturer’s protocol [54]. The sample was completely immersed in equal volumes of Solutions A and B, and the solution was replaced the following day and soaked at room temperature for 2 weeks in the dark. The tissue was transferred to Solution C at room temperature in the dark for 72 h and then replaced by Solution C again for an additional 24 h. The brain tissues were sliced with a microtome cryostat (Leica, Wetzlar, Germany) into 100- to 200-µm-thick sections at −20 °C to −22 °C. The sections were mounted onto gelatin-coated slides using Solution C. Next, the sections were soaked in staining solution consisting of 1 part of Solution D, 1 part of Solution E, and 2 parts of Milli-Q water for 10 min. They were then washed with Milli-Q water twice for 2 min each. Dehydration steps were performed by passing the slides through 50%, 75%, 95%, and 100% ethanol for 4 min each, and xylene twice for 4 min each. Finally, the sections were mounted with neutral resin (Solarbio, China) and stored in the dark at room temperature.

### 4.7. Proteome Analysis of the Prefrontal Cortex of Mice

#### 4.7.1. Sample Preparation

The samples were homogenized in lysis buffer (2 M NaCl, PBS, 1% protease inhibitor). The lysates were transferred to new tubes followed by 3 cycles of sonication consisting of 5 s of active sonication at 30% amplitude with 15 s incubation periods on ice in between sonication pulses. Then, the brain homogenates were centrifuged (1500× *g*, 10 min), and the supernatants were collected. The protein denaturant (8 M urea, PBS, 1% protease inhibitor) was added to the supernatants, the mixture was centrifuged (30,000× *g*, 4 °C, 30 min), and the pellet was discarded. A BCA kit was used to quantify the protein concentration of each sample. The samples were diluted with 50 mmol/L amine bicarbonate until the urea concentration was lower than 1 mol/L, and then 10 mmol/L DTT was added and incubated at 56 °C for 1 h in the dark. After the cooling step, 20 mmol/L iodoacetamide was added, followed by incubation in the dark for 1 h. Trypsin was added to the samples until the ratio of protein to enzyme was 50:1 (*w*/*w*) and incubated overnight at 37 °C.

The SPE cartridge was used to concentrate and desalt the protein. First, the SPE cartridges were activated with acetonitrile and rinsed twice with 0.5% TFA (*v*/*v*). The samples were added to SPE cartridges and then washed twice with 0.5% TFA (*v*/*v*). Next, the cartridges were washed with 200 µL Solution Ⅰ (40% acetonitrile, 0.5% TFA, *v*/*v*) and 200 µL Solution Ⅱ (60% acetonitrile, 0.5% TFA, *v*/*v*) in sequence. The eluates were collected from elutions and then lyophilized and stored at −80 °C. The lyophilized samples were dissolved in a 0.1% formic acid (*v*/*v*) in water and centrifuged at 16,000× *g* for 30 min, and the supernatants were transferred into a new tube using NANODROP to quantify the protein concentrations of the supernatants.

#### 4.7.2. Instruments and Analytical Conditions

A UHPLC-Q-Exactive mass spectrometer (Thermo Scientific, Waltham, MA, USA) was used in the experiments. A gradient elution consisted of mobile phase A (98% H_2_O + 2% acetonitrile + 0.1% FA) and mobile phase B (98% acetonitrile + 2% H_2_O + 0.1% FA), which was applied with the following program: 2% B (0 min) ~ 6% B (0. 1 min) ~ 25% B (80 min) ~ 40% B (110 min) ~ 80% B (115 min) ~ 80% B (125 min). The peptide fragments were separated on a capillary column (150 mm × 100 μm) and a constant flow of 600 nL/min.

#### 4.7.3. Bioinformatics Analysis

Protein quantitation was performed using MaxQuant software. The label-free quantification (LFQ) intensity values were log2 transformed. Only unique peptides ≥ 2 were considered reliable proteins, and reverse peptides and keratins were removed from the analysis. Missing values were imputed based on a normal distribution. Proteins were considered to be differentially expressed when the *p* value for the difference between groups was less than 0.05 (*p* < 0.05).

### 4.8. Western Blotting

Equal quantities of protein (30 µg) were subjected to 10% SDS-polyacrylamide gel electrophoresis and transferred to PVDF membranes (Millipore, Burlington, MA, USA). Membranes were blocked in 5% BSA at room temperature for 1 h. Then, the membranes were incubated with the following primary antibodies overnight at 4 °C: rabbit HDAC1, rabbit HDAC2, rabbit HDAC3 (1:1000, Cell Signaling Technology, Danvers, MA, USA, 65816), rabbit IP3R (1:2000, Abcam, ab108517), rabbit p-CaMKⅡ (1:1000, Cell Signaling Technology, 12716), rabbit CaMKⅡ (1:1000, Proteintech, 13730-1-AP), rabbit ITPKA (1:1000, Proteintech, 14270-1-AP), and rabbit β-actin (1:2000, Proteintech, 20536-1-AP). The PVDF membranes were washed three times with 1× TBST and incubated with HRP-linked secondary antibody (Beyotime Biotechnology, Shanghai, China) at room temperature for 1 h. Finally, these membranes were incubated in an Amersham ECL Western Blotting Detection Kit (GE Healthcare Life Sciences, Chicago, IL, USA) for 30 s–2 min, and the signals were visualized by exposure to Quantitative One Image Analysis (Bio-Rad, Hercules, CA, USA).

### 4.9. Ca^2+^ Assay

The contents of serum calcium were measured by a calcium assay kit (Nanjing Jiancheng Bioengineering Institute, Nanjing, China), following the manufacturer’s instructions. Briefly, 250 µL reagent consisting of 1 part Solution I and 2 parts Solution II was added to the samples and incubated for 5 min. The OD value was measured at 610 nm using a microplate reader (Thermo Fisher Scientific, Waltham, MA, USA).

### 4.10. Statistical Analysis

All values of different groups are expressed as the mean ± SD. The statistical analyses were completed with Student’s *t* test or one-way analysis of variance (ANOVA) followed by Tukey’s post hoc test. *p* < 0.05 was considered statistically significant using Prism 8 (GraphPad Software, La Jolla, CA, USA) and Microsoft Office 2019 (Microsoft, Redmond, WA, USA).

## 5. Conclusions

In conclusion, BG45 could significantly upregulate the expression levels of the synapse-related protein SYT-1 and the cytoskeletal protein NF-L in primary cortical neurons. In vivo, BG45 alleviated damage to dendritic spines and the reduction in synaptic plasticity-related proteins, and reduced the deposition of Aβ, further improving learning and memory function. Further studies found that BG45 might alleviate the imbalance of calcium homeostasis and upregulate synapse-related proteins through the CaMKII/ITPKA/Ca^2+^ pathway, thereby possibly improving synaptic plasticity in the early stage of the AD model. The protective effect and mechanism of BG45 on AD provide a theoretical basis for new strategies for the early treatment of AD.

## Data Availability

Data is available within the article.
